# An Automatic Segmentation and Classification Framework Based on PCNN Model for Single Tooth in MicroCT Images

**DOI:** 10.1371/journal.pone.0157694

**Published:** 2016-06-20

**Authors:** Liansheng Wang, Shusheng Li, Rongzhen Chen, Sze-Yu Liu, Jyh-Cheng Chen

**Affiliations:** 1 Department of Computer Science, Xiamen University, Xiamen 361005, China; 2 Department of Biomedical Imaging and Radiological Sciences, National Yang-Ming University, Taipei 112, Taiwan; Tianjin University, CHINA

## Abstract

Accurate segmentation and classification of different anatomical structures of teeth from medical images plays an essential role in many clinical applications. Usually, the anatomical structures of teeth are manually labelled by experienced clinical doctors, which is time consuming. However, automatic segmentation and classification is a challenging task because the anatomical structures and surroundings of the tooth in medical images are rather complex. Therefore, in this paper, we propose an effective framework which is designed to segment the tooth with a Selective Binary and Gaussian Filtering Regularized Level Set (GFRLS) method improved by fully utilizing three dimensional (3D) information, and classify the tooth by employing unsupervised learning Pulse Coupled Neural Networks (PCNN) model. In order to evaluate the proposed method, the experiments are conducted on the different datasets of mandibular molars and the experimental results show that our method can achieve better accuracy and robustness compared to other four state of the art clustering methods.

## 1 Introduction

The tooth is one of the most important structures in the human mouth. There are a lot of diseases with the tooth, and vertical root fracture (VRF) is a severe disease in human tooth. VRF is defined as a longitudinal fracture confined to the root that usually begins on the internal canal wall and extends outward to the root surface [1]. VRF is a common complication in root canal-treated teeth [2, 3]. This leads to major damage to the periodontium. There exists substantial clinical evidence that VRF also generates a vertical destructive lesion [4, 5]. As pointed in [5], the damage involves both the soft tissues and the adjacent alveolar bones. VRF is a serious threat to the tooth’s prognosis during or after root canal treatment [6].

The diagnosis of VRF often occurs years later by using conventional periapical radiographs. However, recent studies have been shown that the detection of these fractures gets earlier benefiting from Cone-beam computed tomography (CT). Therefore, accurate diagnosis of VRFs can be treated by extraction of teeth from CT images [6]. In our study, we use a Micro Computed Tomography (MicroCT) to collect the tooth image data.

Nowadays, finite element analysis (FEA) is considered as an effective method in endodontic education, training, and treatment. FEA includes a computer model of a material or design that is stressed and analyzed for specific results. In [7], Lertchirakarn et al. used finite element models of maxillary and mandibular incisors to analyze stress patterns. Through FEA, the effect of different ferrule heights on stress distribution within a tooth, which is restored with fibre posts and ceramic crown, is evaluated [8]. In [9], Jones et al. developed a validated 3D finite element method of the movement of a maxillary incisor tooth.

In the circumstances, FEA also can be used on natural and VRF teeth in nonendodontically treated teeth. And then the stress analysis has important accessory diagnostic value for dentistry [10]. During the FEA, setting material properties for each zone is an essential step. In this case, a single tooth can be divided into three parts consisting of enamel, dentin and pulp. However, directly using FEA on the original MicroCT datasets without any processing is infeasible because of complexity of the datasets. Usually, the experienced dentists manually segment and label the different parts of the tooth with different gray value. After that, the labeled images are used as input of FEA. But in practice, manual delineation is a time consuming task. Utilizing computer aided technology will greatly help doctors to obtain the labeled images and reduce their workload. And with a good processing and classification of the original MicroCT datasets, the FEA of the tooth should be more accurate, which is significant to accessory evaluation.

**Fig 1:**
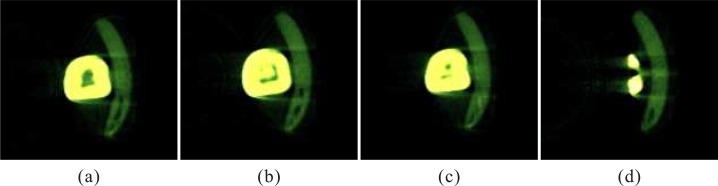
(a), (b), (c) and (d) standing for different cross-sectional slices, respectively. (a) the bracket connected to the tooth. (b) three structures of the tooth. (c) two structures of the tooth. (d) one structure of the tooth.

However, accurate segmentation and classification of the tooth are challenging tasks stemming from the following aspects [11, 12]. (1) MicroCT is a non-destructive imaging technique using X-rays to create cross-sections images, which is seen as a valuable tool in endodontic research [13]. But the MicroCT datasets contains very complicated noisy and artifacts unrelated to the desired object. The gray value of noise is similar to the pixel value of the tooth; (2) due to single tooth, a bracket is needed under the tooth during scanning, which causes the MicroCT data including the bracket, and its close connectivity to the tooth greatly influences segmentation (see Fig 1(a)); and (3) the anatomical structures of tooth are quite complicated, consisting of enamel, dentine and pulp cavity, which leads to a problem that not all structures appear at the same time in the same slice and sometimes there are only one or two structures in the image (as illustrated in Fig 1(b)–1(d)).

The aim of this study is to propose a robust framework for segmentation and classification of different anatomical teeth structures from MicroCT images in both accuracy and efficiency. Instead of direct utilization of GFRLS method, improved GFRLS makes full use of 3D information to generate accurate segmentation results, closely followed by a set of image processing methods. After that, by modifying the pulse output and exploiting spatial adjacency proximity, the PCNN model is improved to be suitable for the complex classification of teeth structures. Our experiments are conducted on sufficient tooth MicroCT datasets and the experimental results are compared to other four state of the art clustering methods with quantitative analysis.

The contributions of this paper reside in the following aspects: (1) we propose a robust and effective framework to automatical categorize structures of the tooth; (2) the proposed method is designed for classification of tooth structures, which can also be extended to other classification tasks; (3) the proposed method enables more accurate and efficient clinical application, such as stress analysis.

The rest of the paper is organized as follows. In Section 2, we review the previous works. The whole framework and the details of our proposed method are described in Section 3. Section 4 shows our experimental set-up and results, which are discussed subsequently. And Section 5 concludes the paper.

## 2 Background

### 2.1 GFRLS Method

Level set is a classical method that utilize partial differential equations (PDEs) and has been applied in medical images, which provides an implementation of an active contour method based on regions or edges to drive the zero level curve towards the object boundary. The GFRLS is one of level set methods and derived from the idea of Chan and Vese (C-V) model and Geodesic Active Contour (GAC) model. GFRLS method is considered as a region-based active contour model, which shares the advantages of the C-V and GAC models. The method is able to control the direction of evolution. When the initial contour is set inside the desired object, it can expand to the object’s boundary. And the initial contour can shrink if the contour is outside the desired object.

Next, we introduce GFRLS in detail. Let Ω be a bounded open subset of *R*^2^, *C*(*p*): [0, 1] → *R*^2^ be a parameterized curve in Ω and *I*: [0, *a*] × [0, *b*] → *R*^+^ be the given image. Image segmentation can be regarded as minimizing the following energy functional [14]:

$$E\left(C\right)=\int_{0}^{1}{\left|C^{{}^{\prime }}\left(p\right)\right|}^{2}dp+\lambda \int_{0}^{1}g^{2}\left(\left|\nabla I\left(C\left(p\right)\right)\right|\right)dp,$$ (1)

However, this model relies on parameters of curve. And in [15], Caselles et al. proposed the GAC model being formulated by minimizing the following energy functional:

$$E\left(C\right)=\int_{0}^{1}g(|\nabla I\left(C\left(q\right)\right)|)|C^{{}^{\prime }}\left(q\right)|dp,$$ (2)

where *g* is an edge stopping function (ESF) to stop the evolution when the contour on the desired object boundaries. It is usually defined as:

$$g\left(\nabla I\right)=\frac{1}{1+{|\nabla G_{\delta }*I|}^{2}}$$ (3)

where *G* denotes a Gaussian kernel with standard deviation *δ* and “*” is a convolution operation.

Using the steepest-descent method to process Eq 2, we can get the formulation:

$$\frac{\partial C}{\partial t}=g(|\nabla I|)\kappa \vec{N}-\left(\nabla g\cdot \vec{N}\right)\vec{N},$$ (4)

where *κ* is the Euclidean curvature of the curve and $\vec{N}$ denotes the unit inward normal.

However, the above GAC model has a drawback when there exists sunk part in the the desired object. In this case, the GAC model cannot segment the object correctly. Therefore, a convergent force is added to make the direction of evolution toward the inside of the curve. Besides, this force also increase the propagation speed. Then Eq 4 can be improved as:

$$\frac{\partial C}{\partial t}=g(|\nabla I|)\left(\kappa +\alpha \right)\vec{N}-\left(\nabla g\cdot \vec{N}\right)\vec{N},$$ (5)

where *α* is the constant velocity parameter.

Let *ϕ* be the level set image. The corresponding level set formulation of GAC can be defined via Eq 5 as follows:

$$\frac{\partial \phi }{\partial t}=g\left(\right|\nabla \left(div\left(\frac{\nabla \phi }{\left|\nabla \phi \right|}\right)+\alpha \right)+\nabla g\cdot \nabla \phi ,$$ (6)

As is pointed in [16], the GAC model has local segmentation property which influences the segmentation precision. Therefore, GFRLS method combines C-V model so as to take advantage of global information of given images.

Chan and Vese [17] proposed a region-based model (ie. C-V model), which can be consider as a special case of the Munfor-Shah problem [18]. For a given image *I* in domain Ω, Ω is divided by a closed curve *C*, including Ω_1_(inside the curve) and Ω_2_(outside the curve). The C-V model is formulated by minimizing the following energy functional:

$$E\left(c,c_{1},c_{2}\right)=\mu \int_{0}^{1}\left|C^{{}^{\prime }}\left(p\right)\right|dp+\lambda_{1}\int_{\Omega_{1}}(I-C_{1})dxdy+\lambda_{2}\int_{\Omega_{2}}(I-C_{2})dxdy,$$ (7)

where *C*_1_ and *C*_2_ denote the mean value of the intensities inside and outside the contour *C*.

To minimize Eq 7, *C*_1_ and *C*_2_ are as follows:

$$c_{1}\left(\phi \right)=\frac{\int_{\Omega_{1}}I\cdot H\left(\phi \right)dx}{\int_{\Omega_{1}}H\left(\phi \right)dxdy},$$ (8)

$$c_{2}\left(\phi \right)=\frac{\int_{\Omega_{2}}I\cdot \left(1-H\left(\phi \right)\right)dx}{\int_{\Omega_{2}}\left(1-H\left(\phi \right)\right)dxdy},$$ (9)

where the “*H*(*ϕ*)” is the Heaviside Function written as:

$$H_{\varepsilon }\left(z\right)=\frac{1}{2}\left(1+\frac{2}{\pi }arctan\left(\frac{z}{\varepsilon }\right)\right),$$ (10)

GFRLS method combines the C-V model to construct a region-based signed pressure force (SPF) as follows:

$$spf\left(I\left(x\right)\right)=\frac{I\left(x\right)-\frac{c_{1}+c_{2}}{2}}{max(|I\left(x\right)-\frac{c_{1}+c_{2}}{2}|)},x\in \Omega ,$$ (11)

The SPF function returns values in the range of [-1,1], which decides the direction of evolution. It adjusts the signs of the pressure forces inside and outside the region of interest so that the curve expands or shrinks.

And then in place of the ESF in the Eq 6, the level set formulation of the GFRLS is as follows:

$$\frac{\partial \phi }{\partial t}=spf\left(I\left(x\right)\right)\cdot \left(div\left(\frac{\nabla \phi }{\left|\nabla \phi \right|}\right)+\alpha \right)\left|\nabla \phi \right|+\nabla spf\left(I\left(x\right)\right)\cdot \nabla \phi ,x\in \Omega$$ (12)

However, re-initialization of signed distance function (SDF) is required in the evolution of traditional level set function. And it is not easy to decide when and how to use re-initialization. Furthermore, re-initialization is time consuming. To address this problem, a Gaussian filter is used to regularize the selective binary level set function after each iteration in GFRLS method. Therefore, the term $div(\frac{\nabla \phi }{\left|\nabla \phi \right|})$ can be removed. Moreover, GFRLS model incorporates global static information to avoid leakages, so the term ▽*spf*(*I*(*x*)) ⋅ ▽*ϕ* is also unnecessary. Finally, the level set formulation of the GFRLS can be written as follows:

$$\frac{\partial \phi }{\partial t}=spf\left(I\left(x\right)\right)\cdot \alpha \left|\nabla \phi \right|,x\in \Omega$$ (13)

### 2.2 PCNN Model

PCNN is an neural model to simulate the mechanism of cat’s visual cortex, which was proposed by Eckhorn et al. [19]. In [20–22], Johnson et al. adapted the Eckhorn model to image processing. And it has been shown that PCNN is widely used in the field of image processing [23] such as image segmentation, pattern recognition [24], edge detection, image enhancement, etc. In this study, we apply PCNN as a clustering method that classifies the different structures of tooth.

**Fig 2:**
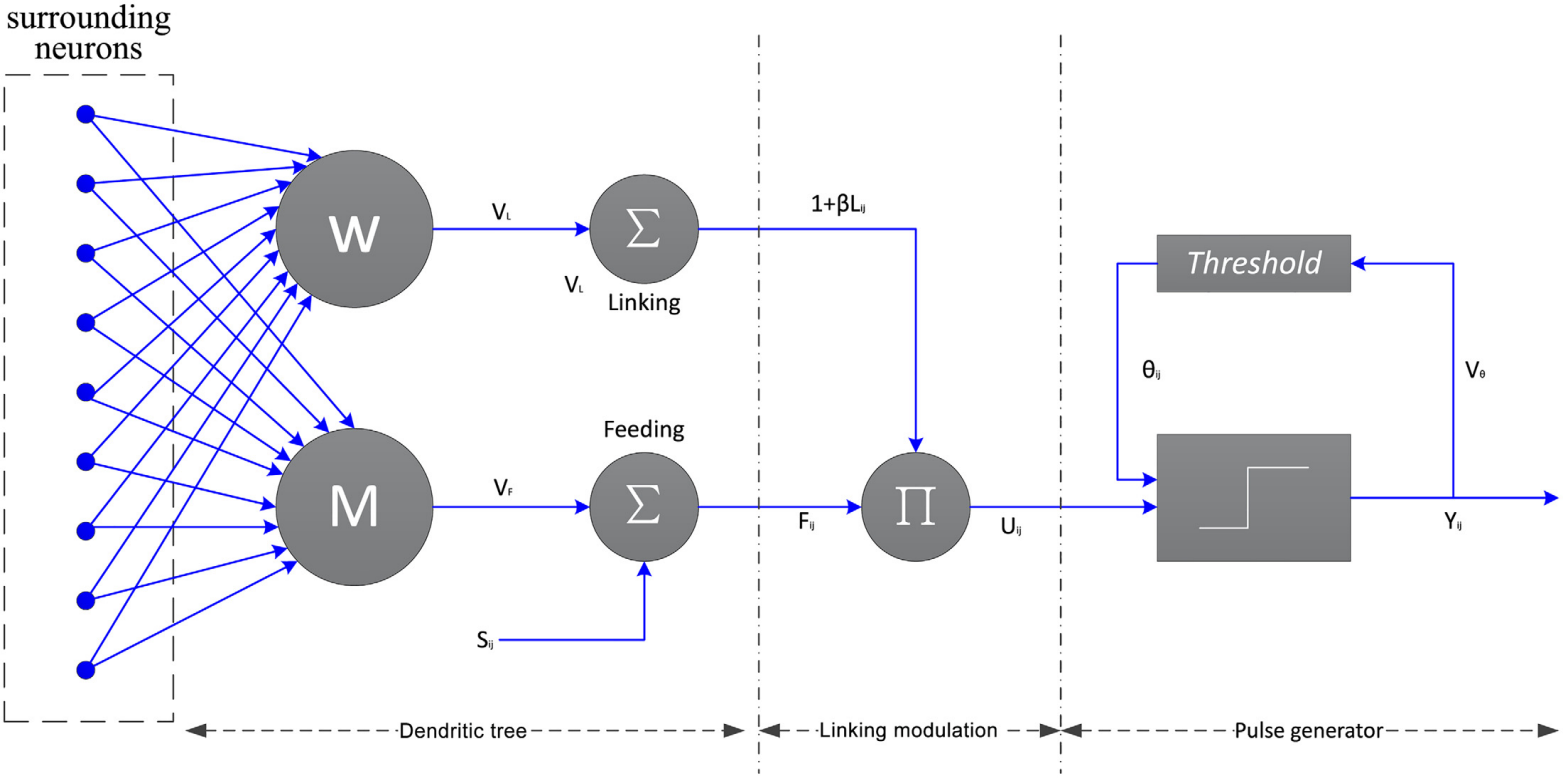
The structure of PCNN model.

As shown in Fig 2, a typical PCNN neuron consists of three parts: the dendritic tree, the linking modulation, and the pulse generator [25]. Each neuron receives signals from both external stimulus *S*_*ij*_ and other neurons. The dendritic tree is used to receive the inputs from two kinds of receptive fields, i.e., the liking and feeding [26]. Also, the two kinds of receptive fields are called two channels. The signals reach the neuron through the two channels: *F*_*ij*_ is the feeding channel and *L*_*ij*_ represents the linking channel. The feeding channel receives local stimulus from the output of surrounding neurons and external stimulus, while the linking only receives local stimulus. In the modulation field, signals from the two kinds channels are integrated with a nonlinear way into the internal activity *U*_*ij*_ which represents the internal state of the neuron. The last field is in the charge of the pulse generating activity-firing [27]. A dynamic threshold *θ*_*ij*_ is utilized to control the firing event so that it is a step function. The essential procedure of PCNN is firing. The neuron fires when the value of *U*_*ij*_ associated with it is equal to or larger than that of the *θ*_*ij*_. if a neuron fires, the the value of corresponding threshold would be set to a very high value. if the neuron does not fire, the value of threshold associated with it would decay exponentially until it is smaller than the neuron’s internal activity, which then makes the neuron fire.

The PCNN model can be described by the following five equations:

$$F_{{}_{ij}}\left(n\right)=e^{-\alpha_{F}\Delta_{t}}F_{ij}\left(n-1\right)+S_{ij}+V_{F}\sum_{k,l}M_{ijkl}Y_{kl}\left(n-1\right),$$ (14)

$$L_{{}_{ij}}\left(n\right)=e^{-\alpha_{L}\Delta_{t}}L_{ij}\left(n-1\right)+V_{L}\sum_{k,l}W_{ijkl}Y_{kl}\left(n-1\right),$$ (15)

$$U_{{}_{ij}}\left(n\right)=F_{{}_{ij}}\left(n\right)\left(1+\beta L_{{}_{ij}}\left(n\right)\right),$$ (16)

$$\theta_{{}_{ij}}\left(n\right)=e^{-\alpha_{\theta }\Delta_{t}}\theta_{ij}\left(n-1\right)+V_{\theta }Y_{ij}\left(n-1\right),$$ (17)

$$Y_{ij}\left(n\right)=step\left(U_{{}_{ij}}\left(n\right)-\theta_{{}_{ij}}\left(n\right)\right).$$ (18)

Each neuron is denoted with indices (*i*, *j*) and (*k*, *l*) refers to its neighboring neurons. *S*_*ij*_, *F*_*ij*_, *L*_*ij*_, *U*_*ij*_ and *θ*_*ij*_ are as described before. *M* and *W* are the synaptic weights. *V*_*F*_, *V*_*L*_ and *V*_*θ*_ represent normalizing constants. *β* is the linking strength and Δ*t* refers to time constant, respectively. *α*_*F*_, *α*_*L*_ and *α*_*θ*_ are corresponding decay coefficient. And *n* is the number of iterations varying from 1 to *N*. Here *Y*_*ij*_(*n* − 1) is the previous pulse and *Y*_*ij*_(*n*) the pulse output. Eqs 14 and 15 stand for the dendritic tree. The linking modulation is given by Eq 16. Eq 17 is the dynamic threshold of the neuron. And firing event is determines by the pulse generator given in Eq 18.

## 3 Method

**Fig 3:**
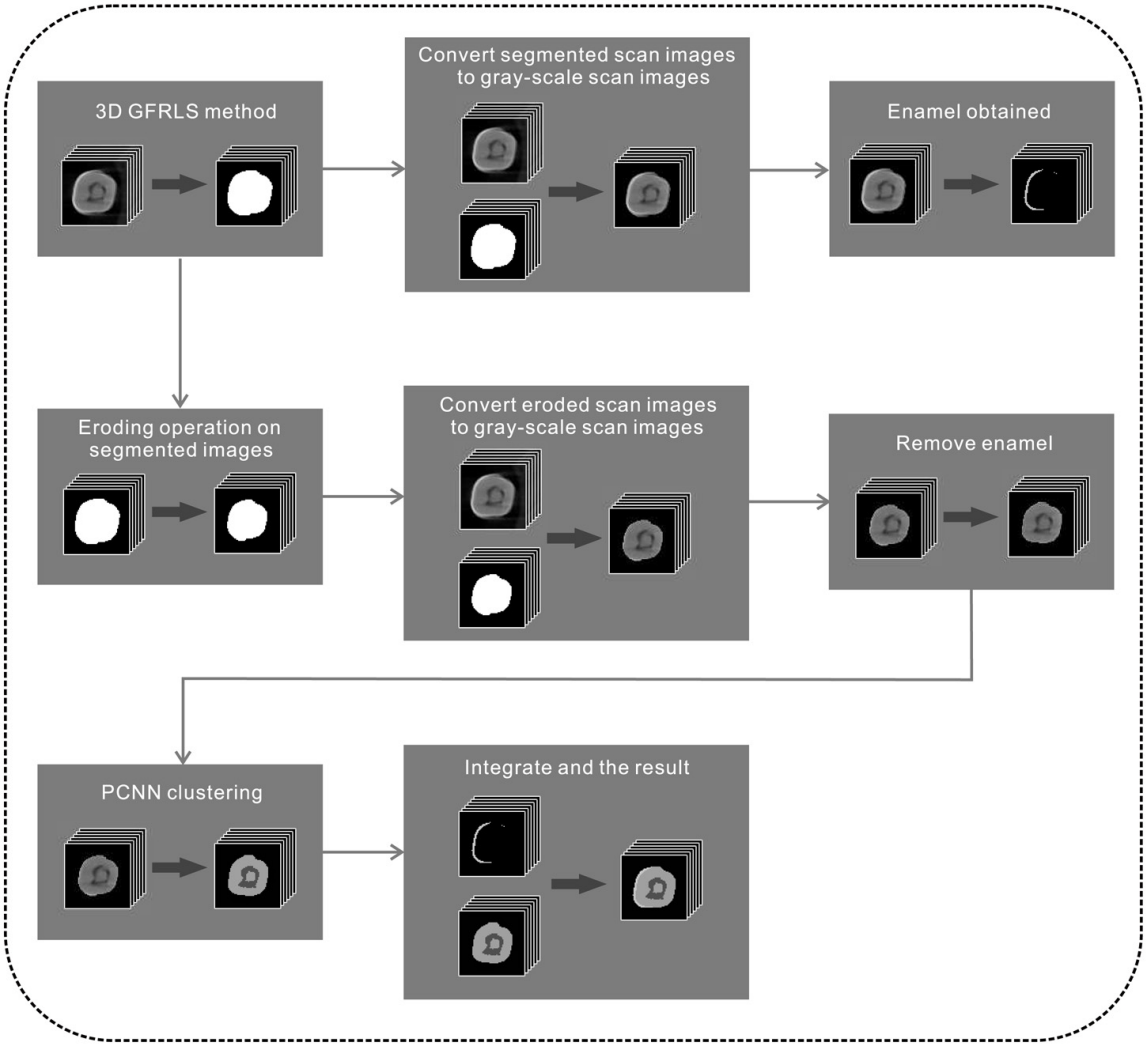
The flowchart of our proposed framework.

In Section 2, we review GFRLS method and PCNN. In this section, GFRLS method based on 3D information and PCNN clustering method are presented. And the detail of our framework of segmentation and classification of tooth is also described. The flowchart of our proposed framework is illustrated in Fig 3. Followed by 3D GFRLS method to segment the tooth, the segmented results in binary images are converted to gray-scale images, in which the enamel is obtained. Also, the eroding operation is applied to remove the noisy on the segmented results in binary images. Then, improved PCNN model is used to classify tooth structures. Finally, the structures are integrated into the resulting images. The institutional review board of National Yang-Ming University approved the study. All data were kept anonymous and confidential and were aggregated for analysis.

### 3.1 3D Segemntation with Improved GFRLS

The MicroCT images include complicated features associated with intensity inhomogeneities, proximity to experimental set-up of similar intensity levels, and weak boundaries of the tooth structures. To address these, GFRLS method is firstly used to process the images. GFRLS has many advantages. (1) GFRLS can easily initialize the level set function by a Gaussian filter; (2) The method utilizes the information inside and outside the contour to control the evolution, which is less sensitive to noise; and (3) A new signed pressure force (SPF) function is used to make the contour efficiently stop at weak or blurred edges.

However, level set methods are usually proposed for two-dimensional (2D) images. Due to the high complexity of the datasets, 2D GFRLS easily segments the undesired parts unrelated to our tasks. 3D segmentation is applied because improved 3D GFRLS fully utilizes 3D global information and it is accurate and efficient. The 3D GFRLS is described as follows.

In Eq 13, let Ω be a bounded open subset of *R*^3^ in 3-D space and *C* be a parameterized tridimensional surface in Ω. The definition of the function *ϕ*(*x*, *y*, *z*, *t*) is the level set image, which represents the four-dimensional space and when the set of definitions *ϕ*(*x*, *y*, *z*, *t*) = 0 denote the surface. And then the points satisfy the following definition:

$$\left\{\begin{array}{ccc} \phi =0, & & \;\left(x,y,z\right)\;\text{is}\;\text{on}\;\text{the}\;\text{C}\;\\ \phi >0, & & \;\left(x,y,z\right)\;\text{is}\;\text{inside}\;\text{the}\;\text{C}\;\\ \phi <0, & & \;\left(x,y,z\right)\;\text{is}\;\text{outside}\;\text{the}\;\text{C}\; \end{array}\right.$$

**Fig 4:**
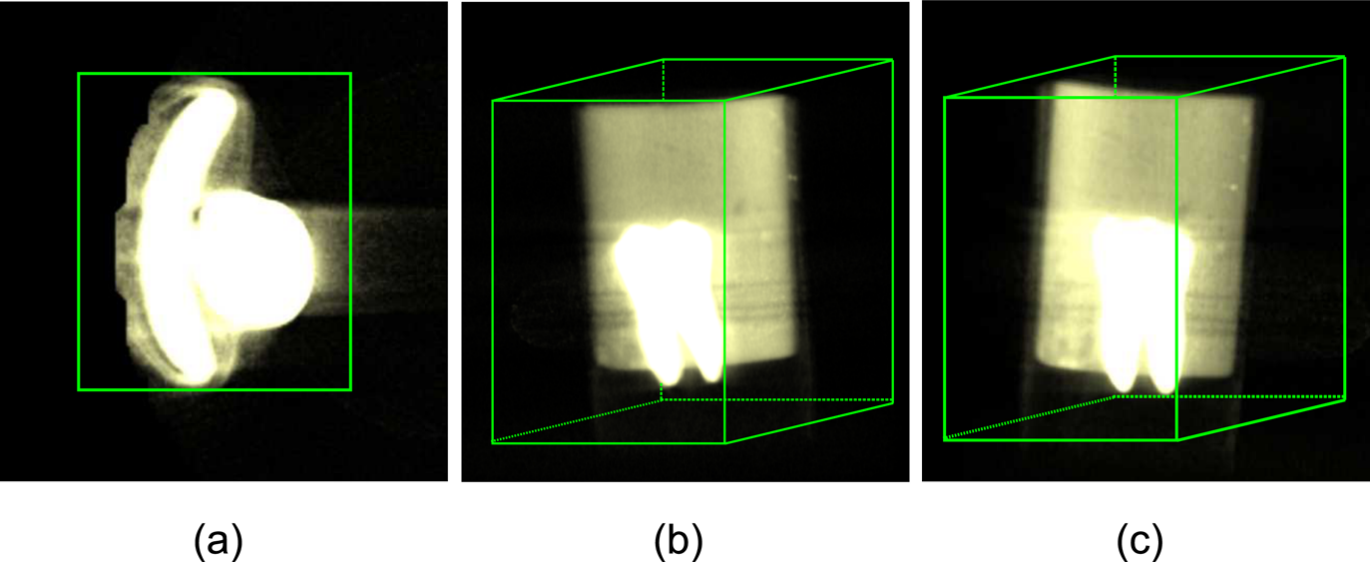
3D initial surface (tooth in volume rendering). (a), (b) and (c) stand for different 3D perspective, respectively.

Under the circumstances, we can initialize the surface closed to 3D volume boundary of MircoCT images sequences. When *ϕ*(*x*, *y*, *z*, *t*) > 0, the points inside the *C* constitute a cuboid encompassing the desired object. Because the intensities of the some structure of tooth are higher than non-tooth parts, we can set *ϕ* inside the contour to 1 and outside to −1. Fig 4 illustrates the initial surface that divides the entire space into two regions (either internal or external to the initial surface) from different 3D perspective. Thus the evolution of the surface is transformed into the evolution of a four-dimensional level set function. In addition, |▽*ϕ*| is calculated in three directions, i.e., *x*, *y* and *z*, which can be written as follows:

$$\left|\nabla \phi \right|=\sqrt{{\left(\frac{\partial \phi }{\partial x}\right)}^{2}+{\left(\frac{\partial \phi }{\partial y}\right)}^{2}+{\left(\frac{\partial \phi }{\partial z}\right)}^{2}}$$ (19)

**Fig 5:**
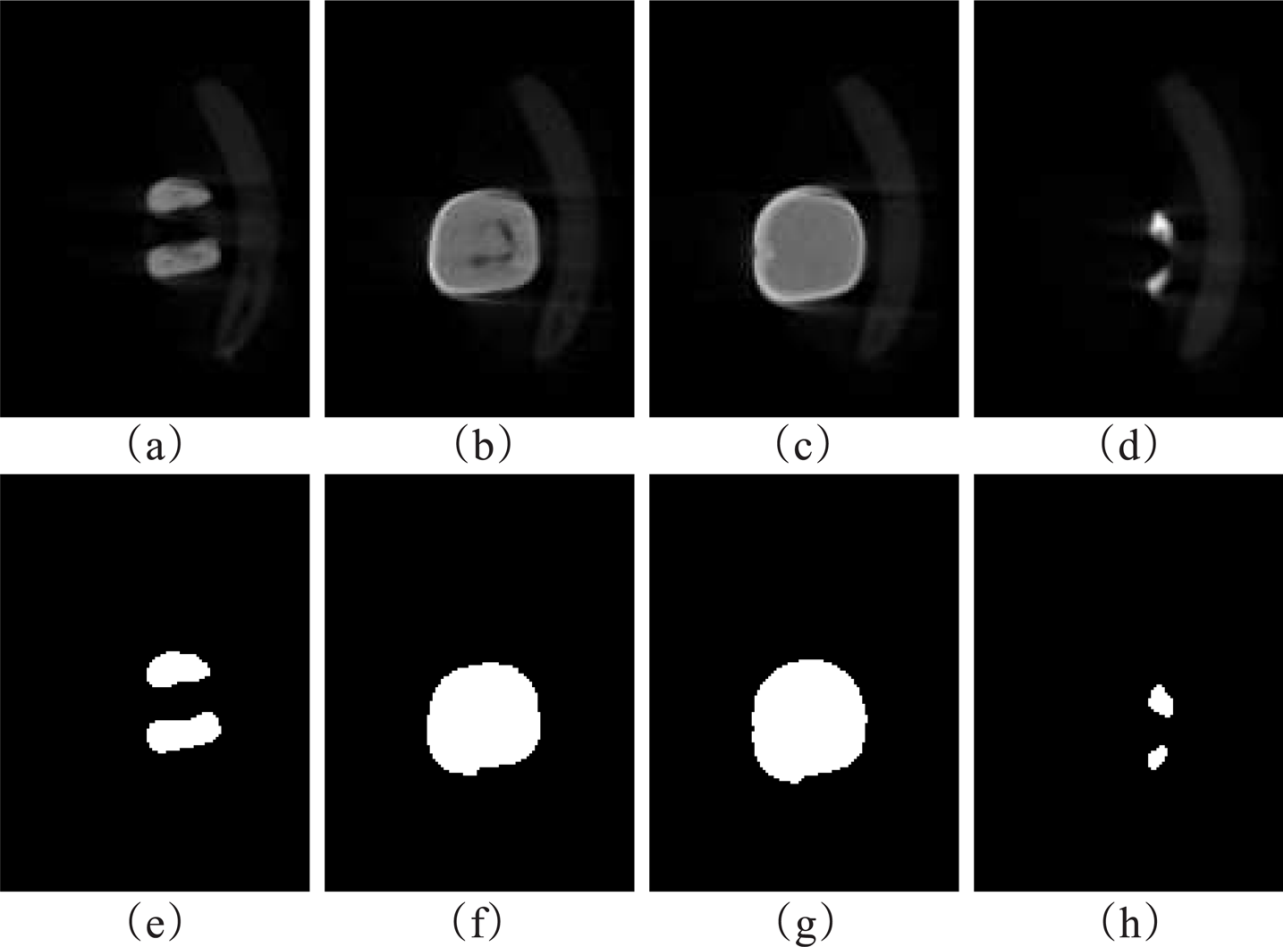
Segmented results by 3D GRFLS method. (a), (b), (c), and (d) are different original MicroCT slices. (e), (f), (g), and (h) are segmented results of the first row.

In the Algorithm 1, the main procedures of the method are shown in detail. Therefore, a closed 3D surface propagates from the initial surface (i.e. a cuboid) toward the tooth boundaries through the iterative evolution of a 4D implicit function. By processing the data by 3D GFRLS method, signed segmented results in binary images are obtained shown in Fig 5.

**Algorithm 1** The procedures of 3D GFRLS method.

1: Initialize the level set function *ϕ*(*x*, *y*, *z*, *t*).

2: Calculate the gradient of *ϕ*(*x*, *y*, *z*, *t*) by Eq 19 and compute *c*_1_(*ϕ*) and *c*_2_(*ϕ*) using Eqs 8 and 9.

3: Update the level set function according to Eq 13.

4: Process the *ϕ*(*x*, *y*, *z*, *t*) by Selective Binary.

5: Regularize the level set function by Gaussian Filter.

6: Check whether the evolution of the level set function converges. If not, return to the step 2.

### 3.2 Tooth Classification with PCNN

After segmenting by 3D GFRLS method, the binary images are achieved which include the tooth (the intensity is 1) and the background (the intensity is 0). Another issue with the original MicroCT images is that in some slice there exists noise which appears with similar image intensity as the dentine at the edge of the enamel or that which has proximity to the pulp on the border of dentin of similar intensity levels. Therefore, morphic erode algorithm is used on the binary images to further removes the noise. And then, by subtraction between original CT data and segmented data, grayscale images of tooth are obtained. A specified threshold is then used to extract the enamel. The steps are clearly outlined in Fig 3. However, dentine and pulp cavity usually have different threshold with regard to different datasets.

Although there exists many unsupervised learning methods used for classifying, PCNN has its own advantages for image classification. First, PCNN model is derived from the observation on cat visual cortex, which is much closer to the human visual processing. Second, the structure of PCNN model is very flexible so that it can be modified according to different kinds of images. Third, PCNN method has been shown the high performance in the literature. Therefore, PCNN is a suitable method to process the classification.

However, the original PCNN model is used to segment images based on similar intensity, producing binary segmented results which weaken the hierarchy of images (i.e classification of images). Nevertheless, in our study, there are different structures of the tooth, and our goal is to classify different structures labeled by several gray values. Therefore, the original PCNN model should be modified for our application, not only reserving the perfect property for segmentation but also serving as classification. The pulse output Eq 18 can be modified as follows:

$$\left\{\begin{array}{c} \xi (n)=U(n)-\theta (n)\\ G_{ij}=\sum_{r}\sum_{t}|\xi_{ij}\left(n\right)-\xi_{i+r,j+t}\left(n\right)|\\ Y_{ij}=\left\lceil \frac{\xi_{ij}\left(n\right)}{\max\xi (n)}\times k\right\rceil \end{array}\right.$$ (20)

where *U*(*n*) is matrix internally activated in n-th iteration, *θ*(*n*) is corresponding threshold matrix, and *D*(*n*) is the difference between *U*(*n*) and *θ*(*n*). *G*_*ij*_ represents the variations of the difference between neuron’s internal activation and the corresponding threshold in 3 × 3 window in which index (*i*, *j*) is the center. $\frac{\xi_{ij}\left(n\right)}{\max\xi (n)}$ serves as normalization and *k* is the parameter about the hierarchies in MicroCT images. The advantages of the improved PCNN model lie in: (1) improve the robustness and effectiveness, because the improved PCNN model makes full use of the information including spatial adjacency proximity for each pixel in 3 × 3 window and similarity in brightness; (2) generate hierarchical result images used for classification, which is appropriate for the datasets.

With regard to image application, in Eqs 14–17 and 20, the indexes *i* and *j* stand for the pixel locations in the input images, i.e. *S*_*ij*_ (1 ≤ *i* ≤ *a*, 1 ≤ *j* ≤ *b*) is the external stimulus referring to the intensity of pixel (*i*, *j*), while *k* and *l* are its neighbor pixels. PCNN is usually performed on 3 × 3 window in order to minimize errors [28]. The 3 × 3 window is denoted by matrix of weight coefficients *W* as follows:

$$W=\left(\begin{array}{ccc} 0.07 & 0.1 & 0.07\\ 0.1 & 0 & 0.1\\ 0.07 & 0.1 & 0.07 \end{array}\right)$$ (21)

**Fig 6:**
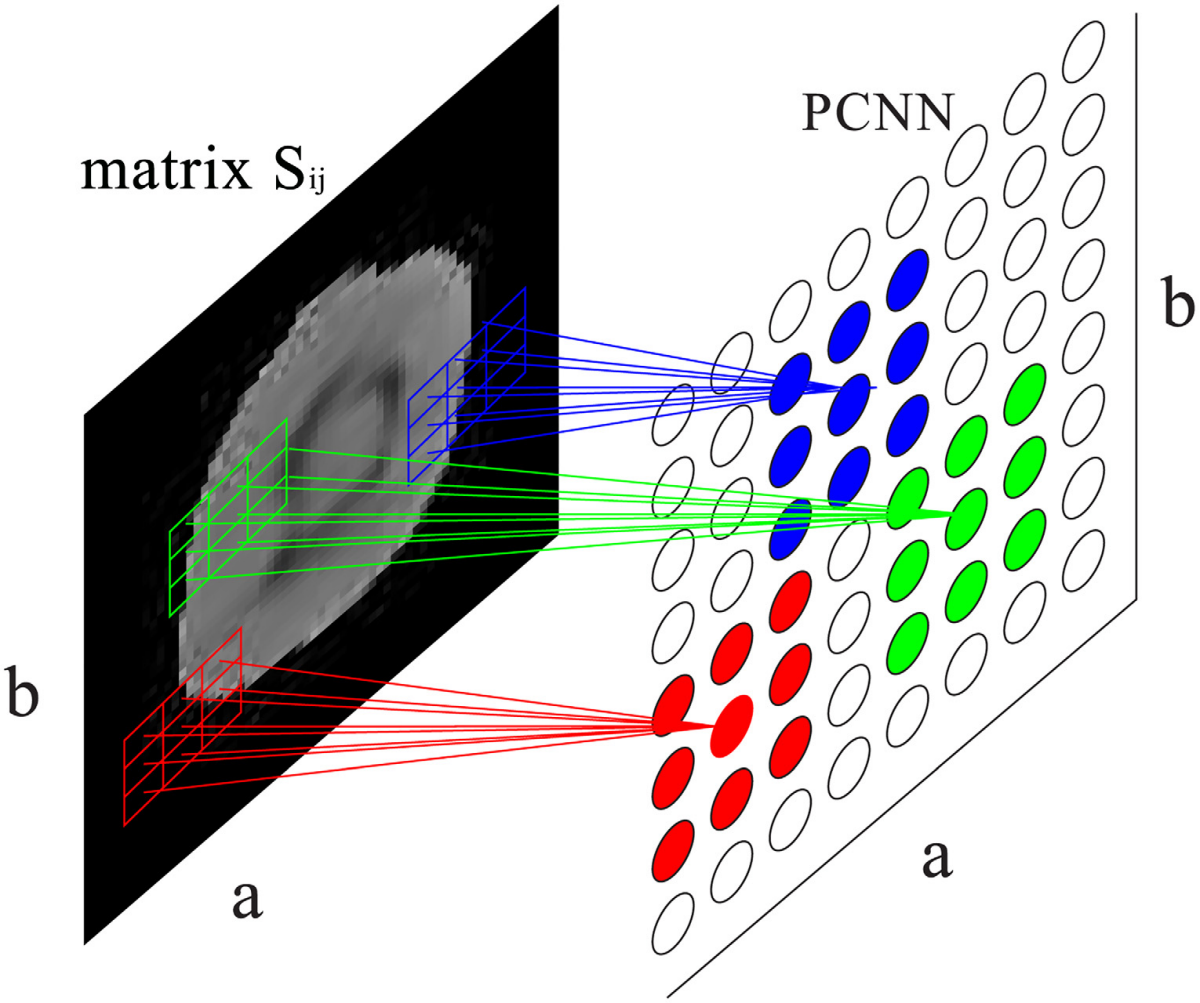
Image processing application of PCNN model. The 3 × 3 grids with different color in the left CT image stand for feeding input of neuron (*i*, *j*), while the 3 × 3 rings in the right image are linking input of neuron (*i*, *j*).

And then, the initial values of all the neurons are set to 1. At the first iteration, the value of interior activity *U*_*ij*_(1) of the neuron is equal to external stimuli *S*_*ij*_. The value of the neuron output *Y* is obtained according to Eq 20. The value of threshold *θ* increases sharply and it will decay exponentially over time. After that, for each iteration, the firing neuron stimulates its adjacent neuron by interacting on the neighboring neurons. If the internal activity of adjacent neuron is larger than or equal to the value of the threshold associated with it, the firing even occurs. Obviously, it is easy to fire when the adjacent neuron has similar intensity with the previous iteration firing neuron. Otherwise, the firing even cannot occur. Therefore, any natural firing neuron will trigger its neighboring neurons firing which have similar intensity. The firing neurons form a cluster of neurons corresponding to a region in which the pixels have similar gray level values in the image. Based on spatial proximity and brightness similarity, the object in the image can be classified. The main procedures of PCNN algorithm are illustrated in detail in the algorithm 2. Fig 6 demonstrates the application of PCNN model for image processing.

**Algorithm 2** The procedures of improved PCNN.

1: Initialize the PCNN network: *S*_*ij*_ is set to the gray level of the corresponding pixel and set the pulse output *Y*_*ij*_, threshold *θ* to 0. Besides, firing frequency *Firate*, which is used to count firing times of each pixel, is also set to 0. And *N* is the total number of iterations

2: **for** 1 to *N* **do**

3: **for** each neuron *S*_*ij*_ in the images **do**

4:  Calculate *L*_*ij*_ by Eq 15;

5:  Compute *F*_*ij*_ according to Eq 14;

6:  Calculate *U*_*ij*_ according to Eq 16;

7:  Acquire *Y*_*ij*_ by Eq 20;

8:  Calculate *θ*_*ij*_ according to Eq 17;

9: **end for**

10:**end for**

11: Classify the images by different neuron output *Y*_*ij*_;

## 4 Experimental Results and Evaluation

### 4.1 Datasets and Ground Truth

**Fig 7:**
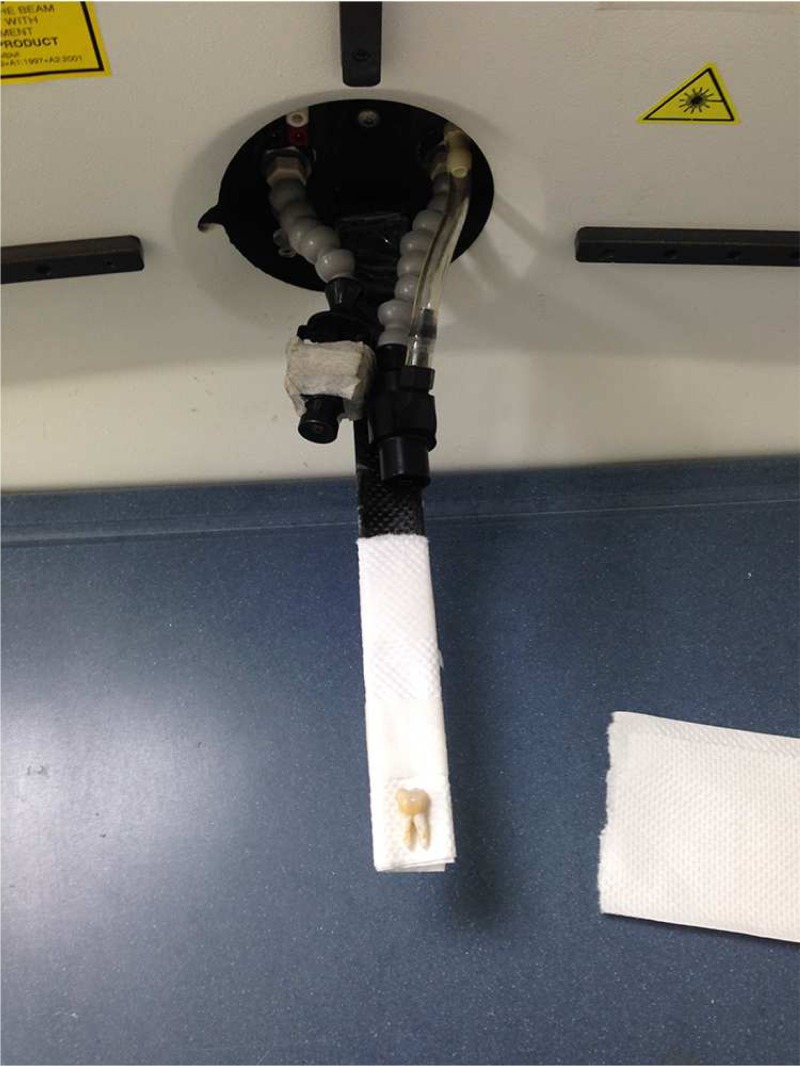
Experimental set-up of MicroCT for tooth scanning.

In order to evaluate the robustness of our method, experiments are conducted on three different mandibular molar MicroCT datasets. Fig 7 shows experimental set-up of MicroCT for tooth scanning. All scans use the 256 × 256 with an in-plain voxel resolution of 1.00 × 1.00 *mm*^2^ and with a slice thickness of 1.00 *mm*. Each dataset represents a single tooth with 256 slices. In order to reduce the calculation, a seed point is set inside the tooth and we set a proper block radius which can contain the tooth. Therefore, a region of interest (ROI) can be extracted from the original MicroCT images, which contains 280 images in total. And the ground truth is generated by experienced clinical dentists who manually label the different anatomical structures of tooth to identify different regions in a segmented mask on each slice of MicroCT datasets. Each ground truth of the classification consists of four labels (one of labels is background) which are displayed by different gray value so that our results can be compared to the ground truth easily.

### 4.2 Quantitative Validation

**Fig 8:**
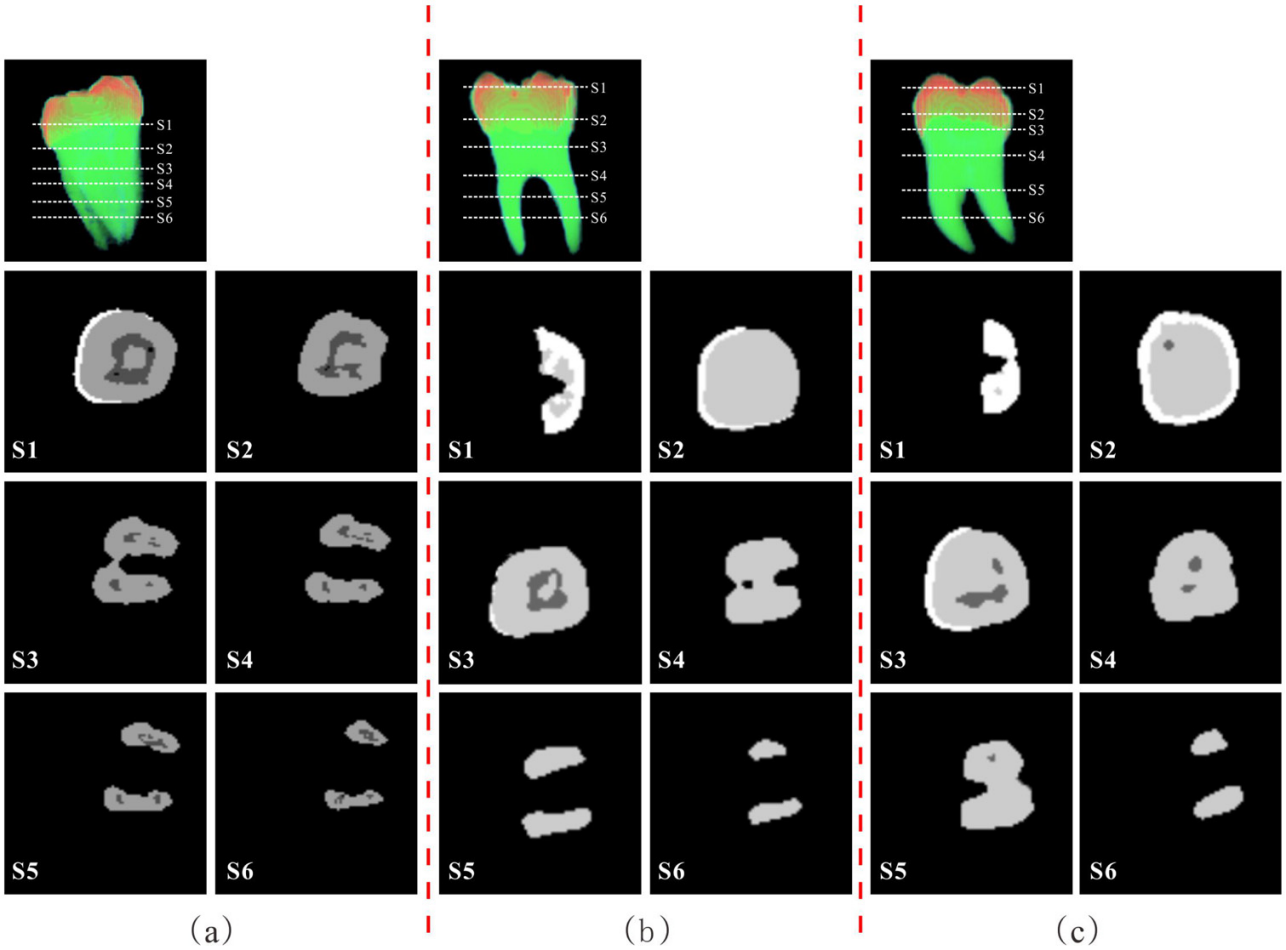
Segmentation and classification results for three MicroCT tooth datasets. For each two column (separated by red dot line), the first top image is the volume rendering of the dataset and the cross-sectional positions in white dot lines. Its segmentation and classification result of each cross-sectional slice is shown in each column.

Our experiments were conducted in Visual Studio 2010 and in Matlab 2013a using Microsoft Windows 7 platform on a CPU of 3.50GHz Intel Core i3-4150 with 16GB of RAM. And our proposed approach is evaluated by calculating the volumes of the different anatomical structures in the single tooth compared to those of the ground truth. Fig 8 shows the final results of the segmentation and classification of the three MicroCT tooth datasets, in which three different grey values represent different tooth structures. It is demonstrated that our method can achieve a robust results in segmentation and classification for single tooth.

**Fig 9:**
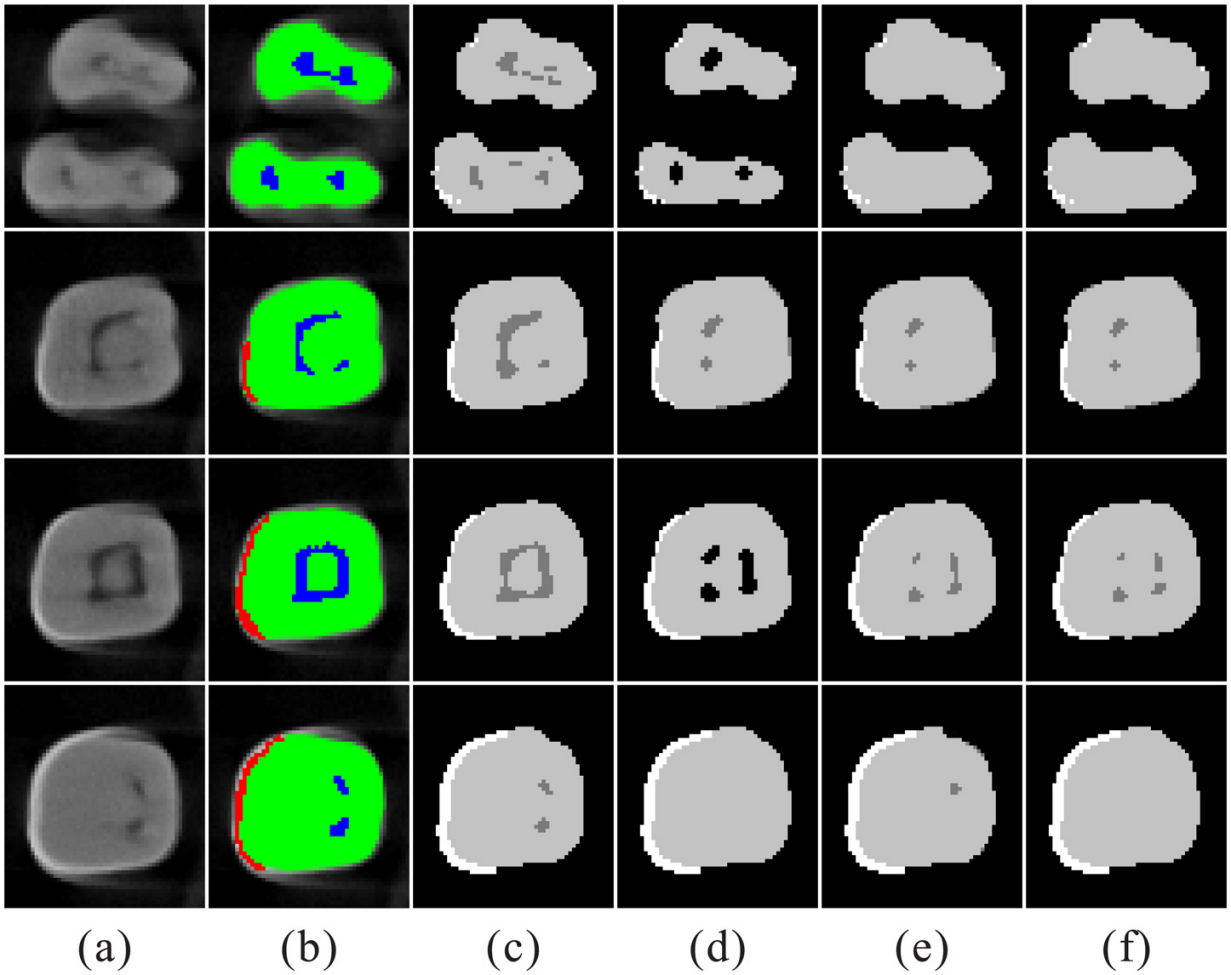
The classification comparison of five methods. Column (a) original MicroCT images. (b) ground truth of classification. (c) results by our method. (d) results by FCM. (e) results by HCA. (f) results by DBSCAN. (g) results by GMMs. In (c), (d), (e), (f), and (g), white color means enamel; black color means background; grey color means dentine; dark grey color means pulp.

Our experimental results are compared with classical clustering methods, i.e., Fuzzy c-means clustering (FCM) [29], hierarchical cluster analysis (HCA) [30], Density-based spatial clustering of applications with noise (DBSCAN) [31], and Gaussian mixture models (GMMs) [32]. All experimental results are quantitatively evaluated by the volume of different structures of tooth. Fig 9 shows that our achieves better classification results compared to FCM, HCA, DBSCAN, and GMMs. Large regions of the pulp are missing and labelled as the dentine or background in the results of FCM, HCA, DBSCAN, and GMMs. It is suggested that our method has more optimal ability to identify the pulp.

**Fig 10:**
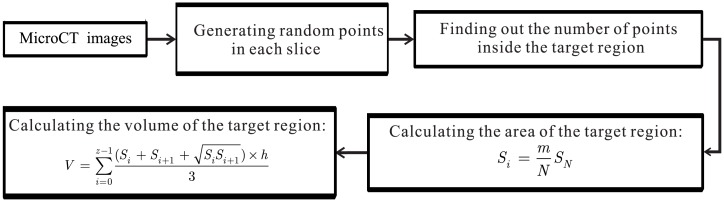
The flowchart of the proposed framework for volume calculation from MicroCT images. *m* is the number of points inside target region; *N* is the number of random points generated; *S*_*N*_ is the area of N-th slice of MicroCT scan; and *S*_*i*_ is the area of target region for each slice.

We define the relative error *E* as the evaluation criteria by the following equation:

$$E=\frac{\left|ComputedVolume-TrueVolume\right|}{TrueVolume},$$ (22)

where *ComputedVolume* and *TrueVolume* denote the volume of the result and the volume of ground truth, respectively. For volume calculation, a robust and accurate method is proposed and implemented that works well on volume calculation from MicroCT images. In the method, Halton low-discrepancy sequences are adopted to calculate the computed and true volume as shown in Fig 10. Based on these, we can quantitatively validate our method in this paper.

**Fig 11:**
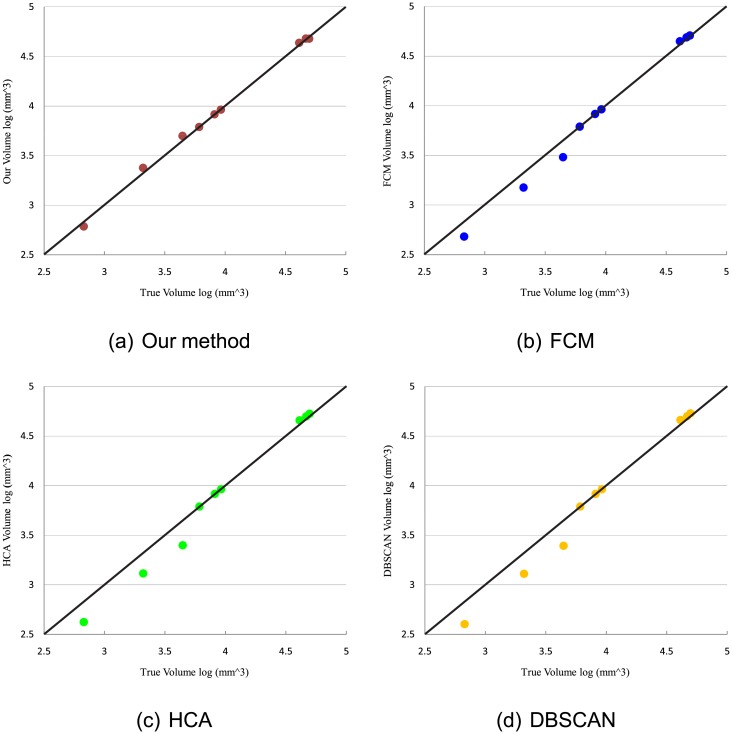
Volume comparison between the results of five methods and ground truth.

**Fig 12:**
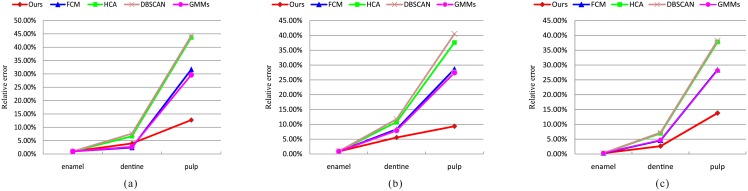
Relative error rate for three datasets.

Fig 11 illustrates the correlation between the results by different methods and ground truth in volume for different structures of tooth. It indicates that the volume distribution of our method is more approximate to that of ground truth than the results of FCM, HCA, DBSCAN, and GMMs. It can be seen that the pulp classified by FCM, HCA, DBSCAN, and GMMs is smaller than that of ground truth, because these four methods easily classify the pulp as the dentine. Table 1 shows the volume results of structures in each data case for four methods and the ground truth. The relative error rate for MicroCT datasets is shown in Fig 12. It is demonstrated that our method presents lower relative error than those four methods. In this figure, the results of the enamel and the dentine have a relatively low error rate compared to pulp. Because pulp is a tiny structure in the tooth, slight difference between the results and the ground truth will greatly influence the relative error.

**Table 1:** The volume results by four methods and the ground truth. The value of each volume is in *mm*^3^. The bold number means the best one for each data case.

Datesets	Ground truth	Ours	FCM	HCA	DBSCAN	GMMs
enamel(case1)	8167.00	**8246.29**	**8246.29**	**8246.29**	**8246.29**	**8246.29**
dentine(case1)	49700.00	47740.80	**50902.60**	53005.00	53500.21	51031.72
pulp(case1)	4437.00	**5001.40**	3030.10	2500.30	2470.60	3120.56
enamel(case2)	6098.00	**6148.80**	**6148.80**	**6148.80**	**6148.80**	**6148.80**
dentine(case2)	41070.00	**43355.80**	44500.10	45500.12	45900.00	44325.70
pulp(case2)	674.00	**610.981**	480.90	420.60	400.95	489.20
enamel(case3)	9221.00	**9194.34**	**9194.34**	**9194.34**	**9194.34**	**9194.34**
dentine(case3)	46650.00	**47886.80**	48800.78	49900.01	50010.53	48856.09
pulp(case3)	2091.00	**2379.74**	1498.09	1300.60	1290.60	1502.36

**Fig 13:**
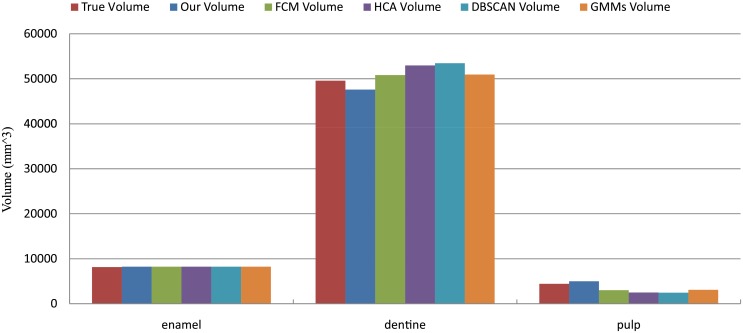
Volume comparison for one of the datasets.

We further evaluate quantitatively our experimental results with a statistical model (i.e. correlation coefficient). The correlation coefficient (*CorrCo*) is defined by the Eq 23, which measures the correlation between our results and true volumes in the range [0, 1], where 1 denotes a perfect fit.

$$CorrCo=\frac{\left|\sum_{i=1}^{n}\left(x_{i}-\bar{x}\right)\left(y_{i}-\bar{y}\right)\right|}{\sqrt{\sum_{i=1}^{n}{\left(x_{i}-\bar{x}\right)}^{2}\;\cdot \;\sum_{i=1}^{n}{\left(y_{i}-\bar{y}\right)}^{2}}}$$ (23)

where *x*_*i*_ and *y*_*i*_ denote two sets of data and *n* is the number of data. $\bar{x}$ and $\bar{y}$ are mean value of *x*_*i*_ and *y*_*i*_, respectively. In our case, let *V*_*enamel*_, *V*_*dentine*_, and *V*_*pulp*_ be vectors including the all computed volume by our method for the three structures (enamel, dentine, pulp) and *V*_*T*_ be a vector containing the corresponding ground truth volumes. Fig 13 shows *V*_*enamel*_, *V*_*dentine*_, and *V*_*pulp*_ compared to *V*_*T*_ in one of MircoCT datasets, which illustrates that volumes computed by our proposed method are closer to those obtained from manual segmentations than those four methods. Although FCM has a comparable classification results of dentine, but it will misclassify the pulp as the dentine. We also evaluate the volume similarity between our method and the ground truth by correlation coefficient. The result by our proposed method has a *CorrCo* of 0.9685, which demonstrates a high conformity between the vectors.

Over all, experimental results show that our method achieves a good classification of the tooth. Furthermore, mean absolute deviation (MAD) [33] is used to estimate the absolute deviations from the ground truth, which demonstrates our proposed method is superior to those four methods as shown in the Table 2.

**Table 2:** Statistical evaluation. MAD of volumes for four different methods.

Ours	FCM	HCA	DBSCAN	GMMs
**728.6343**	1015.127	1569.506	1687.816	1011.089

To further evaluate quantitatively our results in an efficient manner, some more validity indexes-the similarity index (*S*), the sensitivity (*SENS*), and the specificity (*SPEC*)-are also employed. The automatic classifications obtained by different methods (*A*) are compared to the corresponding manual classifications by clinical doctors (*D*). |*A*| is the amount of the set of pixels *A*. Experimental results are quantitatively assessed using the validity indexes calculated as $S=\frac{2|A\cap D|}{\left(|A|+|D|\right)}$, $SENS=\frac{\left|A\cap D\right|}{\left|D\right|}$, and $SPEC=\frac{\left|A\cap D\right|}{\left|A\right|}$. As pointed in [34], an advantage of the similarity index *S* is that it sensitively reflects the variations in shape, size and a strong agreement is indicated with the value of *S* > 0.7. The sensitivity (*SENS*) and specificity (*SPEC*) offer us additional information about how the overlap between *A* and *D* is obtained. For example, if the comparison of *A* and *D* generates a high sensitivity value but a low specificity one, this means the automatic classification is too large. When total overlap is attained, all of these validity indexes are equal to 1.

Table 3 shows the results of five methods based on different validity indexes. Note that our method outperforms other four clustering methods, especially in pulp. This is because *S* of our method is higher than 0.7 and higher than that of those four methods. Specifically, for dentine, the values of the sensitivity are in general higher than those of specificity, since the dentine is over-segmentation, i.e., the pulp is misclassified as the dentine, which is consistent with the problem that the segmentation results of pulp is small. In addition, due to the fact that the pulp is very tiny tooth structure, slight difference between automatic classification result and ground truth will lead to large deviation. Therefore, validity indexes for pulp can not achieve a relatively good outcome. However, our method can still produce satisfying classification results both in dentine and pulp. This is confirmed by the high value of *S* (0.792), sensitivity (0.746) and specificity (0.780) regarding pulp.

**Table 3:** Comparison of different methods based on different indexes.

Method	Similarity index	Sensitivity	Specificity
Ours			
enamel	**0.847**	**0.942**	**0.840**
dentine	**0.895**	0.950	**0.896**
pulp	**0.792**	**0.746**	**0.780**
FCM			
enamel	**0.847**	**0.942**	**0.840**
dentine	0.853	**0.956**	0.813
pulp	0.723	0.617	0.657
HCA			
enamel	**0.847**	**0.942**	**0.840**
dentine	0.829	0.950	0.802
pulp	0.656	0.560	0.648
DBSCAN			
enamel	**0.847**	**0.942**	**0.840**
dentine	0.812	0.951	0.795
pulp	0.642	0.551	0.623
GMMs			
enamel	**0.847**	**0.942**	**0.840**
dentine	0.851	0.952	0.809
pulp	0.715	0.610	0.623

There are some reasons needed to be highlighted. Firstly, improved PCNN model takes advantage of local information of images, i.e., spatial adjacency proximity for each pixel, which can generate robust and efficient classification results. Secondly, FCM and GMMs are sensitive to the initial cluster centers and easily fall into local optimum instead of achieving global optimal steadily. Thirdly, for HCA, when the agglomeration or the division is performed, it can not be modified, which influences the classification results. Fourthly, DBSCAN is not entirely deterministic, and the quality of DBSCAN depends on the distance measure. Thus, our method has more optimal ability to these datasets than those four methods.

## 5 Conclusion

In this study, 3D GFRLS method and improved PCNN method are proposed in our framework to segment and classify the tooth. After cutting out ROI from the original MicroCT images, the 3D GFRLS method is proposed and used to remove the noise and segment the tooth precisely. Then several processing steps are used to further remove unneeded artifacts. We get the final results by employing an improved PCNN. By comparing to those four clustering methods, experimental results show that our proposed method can achieve better accuracy and robustness. Therefore, our method enables a more efficient and accurate way to FEA. We believe our framework will play an effective role in the clinical accessory diagnosis of dentistry.

One of the foreseen improvements of our method is about self-adaption, that is, parameters can be adjusted automatically, since different parameters contribute to the quality of classification. In addition, further applications include different kinds of datasets, such as teeth with periapical lesion, and more datasets are collected for further evaluation in clinical use.
